# Emergency Department Characteristics and Capabilities in Quito, Ecuador

**DOI:** 10.5334/aogh.3129

**Published:** 2021-04-07

**Authors:** Augusto Maldonado, Andrés M. Patiño, Alexis S. Kearney, Diana Tipán, Valerie Chavez-Flores, Michaela Banks, Krislyn M. Boggs, Carlos A. Camargo

**Affiliations:** 1 Department of Emergency Medicine, Hospital General Docente Calderón, Universidad San Francisco de Quito, Quito, Ecuador; 2 Department of Emergency Medicine, Emory University School of Medicine, Atlanta, GA, US; 3 Department of Emergency Medicine, Brown University, Providence, RI, US; 4 Hospital General IESS Quito Sur, Quito, Ecuador; 5 Brown University Warren Alpert Medical School, Providence, RI, US; 6 Louisiana State University School of Medicine, New Orleans, LA, US; 7 Massachusetts General Hospital, Boston, MA, US

## Abstract

**Background:**

Emergency care is an essential part of a health system. Ecuador has recognized emergency medicine as a specialty and has two emergency medicine residency training programs. However, little has been published about emergency department characteristics and capabilities in Ecuador.

**Objective:**

We described the characteristics and capabilities of emergency departments (EDs) in Quito, Ecuador, in 2017, using the National Emergency Department Inventory (NEDI) survey.

**Methods:**

The 23-item survey included questions pertaining to ED characteristics, including: visit volume, physical and administrative structure, clinical capabilities, technological resources, and consult personnel availability. This study included all EDs in Quito operating 24 hours/day, 7 days/week, and serving all patients seeking care. One representative from each ED was asked to complete the survey based on calendar year 2017.

**Findings:**

Thirty EDs met the inclusion criteria, and 26 completed the survey (87% response). The median number of ED beds was 17 (range 2–61). Median annual visit volume was 22,580 (range 1,680 to 129,676). All but two EDs provided care for both children and adults. Cardiac monitors were available in 88% of EDs, CT scanners in 68%, and rooms for respiratory isolation in 31%. Most EDs could manage patients with general medicine (92%), general surgery (92%), and gynecology (88%) emergencies 24/7. Fewer were able to provide hand surgery (45%) and dental (28%) care 24/7. Typical length of stay was 1–6 hours in 65% and >6 hours in 31% of EDs. Half of EDs reported operating at full capacity and 27% reported operating over their capacity. When compared to private EDs, government EDs (public and social security) had a higher mean number of visits per year (50,090 government vs. 13,968 private, p < 0.001), higher mean number of ED beds (36 government vs. 9 private, p = 0.002), and higher length of stay (58% of patient stays > 6 hours in government EDs vs. 86% of patient stays 1–6 hours in private EDs, p = 0.009).

**Conclusions:**

EDs in Quito varied widely with respect to annual visit volume, ability to treat different pathologies 24/7, and resources. Most EDs are functioning at or over capacity, and a substantial number have long lengths of stay. Further research and investment in emergency care could help increase the capacity and efficiency of EDs in Ecuador.

## Background

Ecuador is an upper-middle-income country in northern South America with a population of 17.4 million [1]. Quito is the capital of Ecuador and is located 2,850 meters above sea level, high in the Andes mountain range. It is currently the most populated city in Ecuador, with approximately 2,736,000 total inhabitants [2]. Ecuador experienced significant economic growth in the last two decades with 1.4 million people coming out of poverty [3]. Life expectancy is currently 77 years [4].

Over the last three decades, considerable improvements have been made within the realm of emergency care in Ecuador. In the late 1990s, national and international investment in the healthcare sector increased with the implementation of FASBASE (*Fortalecimiento y Ampliación de los Servicios Básicos de Salud en el Ecuador* or Strengthening and Expansion of Basic Health Services in Ecuador). This program included specific provisions for the improvement of emergency and prehospital care [5]. In 1997, the emergency response system, ECU911, was implemented in the country, further advancing emergency care within Ecuador [5].

The Ecuadorian constitution, established in 2008 after more than a decade of political instability in which the country had seven presidents in a ten-year span (1995–2005), guarantees free and unconditional access to emergency care for life-threatening conditions in both the public and private healthcare sectors [6]. Ecuador has made significant financial and human investments in health care since 2000. Annual health spending increased from US$103 million in 2000 [7] to US$2.570 billion in 2015 (9.2% of GDP) [8]. In 2012, Ecuador’s Ministry of Public Health established the National Directorate for the Standardization of Human Talent in Health (*Dirección Nacional de Normatización del Talento Humano en Salud)*; its mission was to “define, regulate and ensure compliance with standards related to the planning, management, training and development of human talent in health.” The institution’s efforts have focused on restructuring medical education and training, providing scholarships for specialist training, and increasing salaries for health professionals, among other activities [9]. Furthermore, Ecuador has made significant advancements in health infrastructure since 2014, with the construction of Nueva Aurora Gynecological Obstetric Hospital (2014), Hospital Docente Calderón (2014) [10], and Hospital General del IESS Quito Sur (2017), the first new hospitals in Quito in more than 30 years.

The availability of health professionals varies considerably across the country; this inequality is even more pronounced with respect to specialist physicians and dentists. Overall, Ecuador has approximately 20 physicians per 10,000 population [8]. However, the urban zones in metropolitan Quito have an estimated 40 physicians/10,000 residents [11], and the majority of Ecuador’s residency-trained emergency physicians work within the greater Quito area [12].

Emergency Medicine (EM) residency programs began in 1989, and the field was recognized as a specialty in 1993. There are currently two EM residency programs in the country and approximately 300 EM trained physicians nationwide. However, a report determined that there was a deficit of approximately 1,400 EM physicians in 2016. This deficit is expected to be corrected by 2030 as more residents graduate [6]. A recent qualitative study described the current state of emergency medicine in Ecuador cited challenges across multiple sectors, including medical care, working conditions, residency education, leadership, and prehospital care [12]. The authors identified specific areas for improvement and argued in favor of stronger collaboration and advocacy amongst EM physicians.

In May 2019, the World Health Organization called on member states to prioritize the provision of universal emergency care [13]. Research across a broad range of disease processes has demonstrated time and again the impact a systematic approach to emergency care can have on morbidity and mortality. In fact, it is estimated that over 50 percent of deaths in low- and middle-income countries (LMICs) can be addressed by the implementation of systematic prehospital and facility-based emergency care [14]. In Ecuador, the leading causes of death are ischemic heart disease, diabetes, cerebrovascular disease, hypertensive disease, flu and pneumonia, and land transport accidents [8]; this is consistent with the leading causes of death and disability worldwide published by the Global Burden of Disease Study 2019 [15]. Furthermore, it stresses the need for strong emergency care systems to address injuries and the ever-increasing burden non-communicable diseases place on the health care system.

While important advancements have been made, little has been published about emergency departments (EDs) and emergency care in Ecuador. To address this knowledge gap, we used the National Emergency Department Inventory (NEDI) survey to conduct a cross-sectional assessment of the characteristics, capacities, and capabilities of EDs in Quito, Ecuador [16]. The NEDI survey has been used in more than a dozen countries worldwide [16]. Thus, this study not only serves as a benchmark against which Ecuador can track improvements in emergency care capacity over time but also allows for comparison against other countries.

## Methods

### Study Design

This was a cross-sectional, descriptive study of EDs administered via paper survey. Ethical approval was obtained from the Partners Healthcare Institutional Review Board (Boston, USA), and from the Hospital General Docente Calderón Institutional Review Board (Quito, Ecuador). The first page of the survey included information about the survey. Consent was implied by voluntary participation in the survey.

### Study Protocol and Measurements

A list of all health facilities in the city of Quito open during 2017 was obtained from Quito’s Health Department (*Secretaría de Salud*). Facilities with names unlikely to be related to a general hospital or ED were excluded (e.g., facilities whose names included words such as “dental,” “maternal,” “psychiatric,” “surgical,” or “laboratory”). The websites of the remaining facilities were reviewed to identify EDs meeting two inclusion criteria: (1) must provide emergency care 24 hours/day, 7 days/week, and (2) serve all patients seeking care for any emergent complaint. Two local authors, AM and DT, reviewed the final list based on their many years of experience working in emergency care in Quito and agreed on the final list of EDs.

The NEDI survey tool includes 23 questions divided into four categories: ED characteristics, patient experience, capacity, and resources and capabilities. A Spanish version of the survey – from a previous NEDI study in Bogota, Colombia – was used with permission [11]. The survey questionnaire can be found in online Supplement A.

### Survey Administration

One representative from each ED (typically the ED director) was asked to complete the survey based on data from his/her ED for the 2017 calendar year. The survey was initially sent to hospital representatives via email. Unfortunately, no one completed the survey. As a result, the decision was made to administer the survey in person using a paper version. Hospital representatives were contacted via phone and WhatsApp. Contact information was obtained by authors AM and DT through their own social and professional networks. After completion of the survey, we transferred responses from paper forms into a secure online database (LimeSurvey) [12]. Data were collected from April 2018 to July 2019. Follow-up data collection was conducted in November 2019 for 2017 annual ED visit volumes as many participants were unsure of their annual visit volumes during the first round of data collection.

### Data Analysis

Data analysis was performed with Microsoft Excel (2019) and Stata (version 15.1). For the overall data, medians and interquartile ranges (IQR) were calculated for continuous variables; proportions and 95% confidence intervals were calculated for categorical variables. Chi-square tests were used to investigate the association between ED size and length of stay. Emergency departments were then divided into government EDs (public and social security EDs) and private EDs. Means, proportions, and confidence intervals were calculated, and Fisher’s exact test was used to compare variables across the two groups.

## Results

### Sample Selection

**Figure 1:**
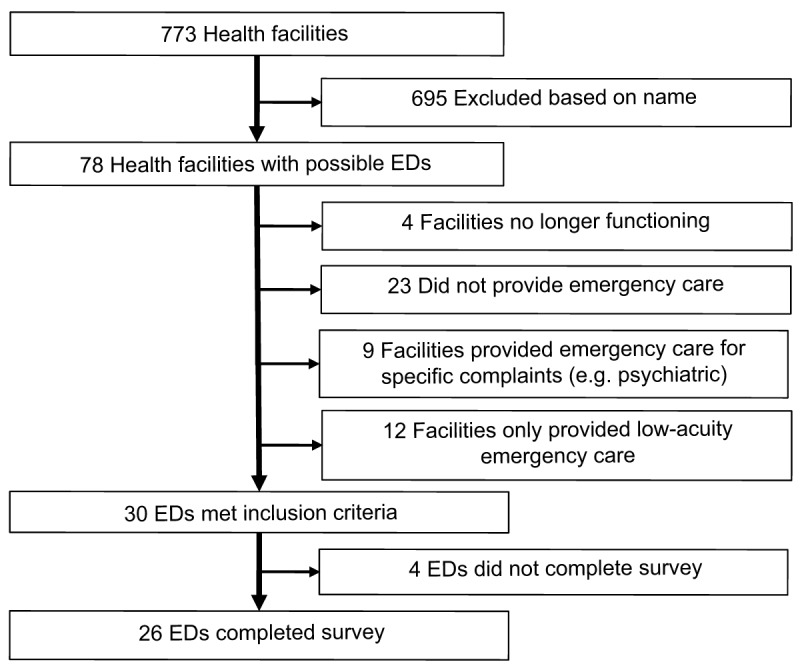
Sample Selection: inclusion/exclusion algorithm used to establish study sample.

**Figure 2:**
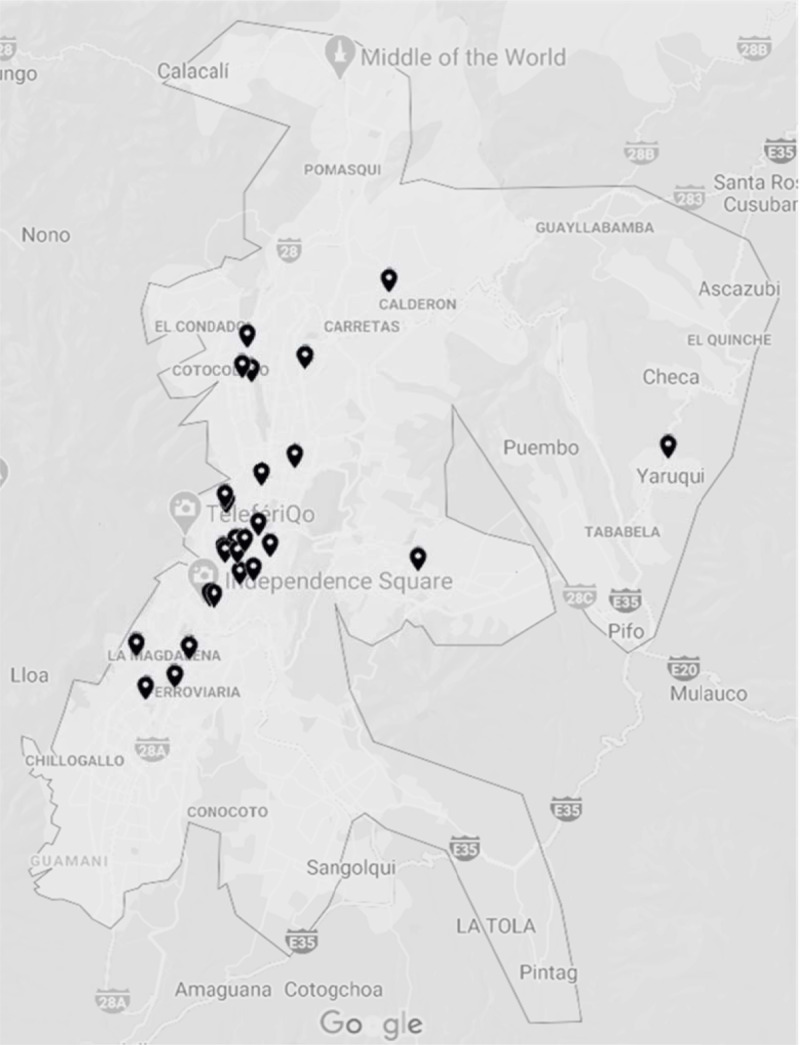
Geographic Distribution of Emergency Departments in Metropolitan Quito.

Quito’s Health Department provided a list of all 773 health facilities in the city, of which 695 were excluded based on name (310 laboratory or diagnostic centers, 195 outpatient centers, 73 specialty centers [37 dental, 5 orthopedics, 5 radiologic, 4 ophthalmologic, 3 neurologic, 3 occupational, 3 gynecologic, 2 rheumatologic, 2 surgical, 2 pediatric, 2 alternative medicine, and 5 from other specialties] 47 individual providers, 33 non-clinical centers, and 27 duplicate names). Seventy-eight facilities were identified as potentially providing emergency care based on the facilities’ names. The websites of the 78 facilities were reviewed and 30 facilities met the inclusion criteria (***Figure 1***). Two exceptions were made to include one ED that only cares for children (age <16 years) and one ED that only treats adults (age ≥16 years), given the prominent role these facilities have in the provision of emergency care in Quito. Twenty-six EDs completed the survey (87% response rate). Of the four facilities that did not answer the survey, two declined to participate and two did not respond to multiple requests. ***Figure 2*** shows the geographic distribution of EDs in the metropolitan area of Quito.

### ED Characteristics

All EDs were open 24 hours/day, 7 days/week, and were physically connected to a hospital. All but two EDs cared for both children and adults; one ED only provided care for children and one ED only provided care for adults. The median number of annual ED visits was 25,898 (IQR 12,043–45,050; range 1,680–129,676). Based on this data, we estimate 336 ED visits per 1,000 population in Quito. The median percent of ED visits by children was 30% (IQR 23–38%, range 0–100%). The median number of ED beds was 17 (IQR 6–33; range 2–61), and the median number of hospital beds was 59.5 (IQR 34.5–221; range 15–610), as shown in ***Table 1***. All but one ED had a contiguous layout, with medical and surgical care provided in the same area. More than half of EDs (54%) reported triaging patients to a specific service within the ED (e.g., medical, surgical, pediatric, ***Table 1***).

**Table 1:** Characteristics of Emergency Departments in Quito, Ecuador (n = 26, 87% response).


	PROPORTION OR MEDIAN	95% CONFIDENCE INTERVAL OR INTERQUARTILE RANGE

**ED Characteristics**		

Contiguous	96%	89–100%

Total visits (n = 26) (median)	25,898	12,043–45,050

Percent of visits by children (n = 20, median)	30%	23–38%

ED beds (n = 26, median)	17	6–33

Triage to service (n = 23)	57%	36–76%

**Patient Experiences in the ED**		

Percent of ED pts arriving by ambulance (n = 23)		

<20	43%	23–64%

20–39	17%	2–33%

40–59	13%	0–27%

Unknown	26%	8–44%

Length of Stay (n = 26)		

<1 hour	4%	0–11%

1–6 hours	65%	47–84%

6+ hours	31%	13–49%

Percent of ED visits leading to admission (n = 24)		

<20	21%	2–32%

20–39	42%	18–57%

40–59	21%	5–37%

Unknown	17%	2–32%

**Resources and Capabilities**		

Nurse in ED 24/7 (n = 26)	100%	–

Physician for ED (n = 25)		

In ED 24/7	96%	88–100%

In Hospital 24/7	4%	0–12%

On call from home 24/7	0%	–

Mechanical ventilator (n = 25)	96%	88–100%

24-hour lab availability (n = 26)	92%	82–100%

Cardiac monitor (n = 26)	88%	76–100%

EMR (n = 26)	73%	56–90%

Dedicated CT (n = 25)	68%	50–86%

Negative pressure room (n = 26)	31%	13–49%


Abbreviations: ED = emergency department; EMR = electronic medical record; CT = computed tomography; n = number of EDs that answered question; Unknown = respondent did not know answer.

### Patient Experiences in the ED

Forty-three percent of respondents stated that <20% of patients arrive to the ED by ambulance, while one-quarter of respondents (26%) were not sure of the percentage of patients who arrived by ambulance. Most EDs (65%) reported patient length of stays of 1–6 hours, while 31% reported stays of over 6 hours. EDs with greater than 55,000 visits per year were more likely to report average stays of 6 hours or more (p = .002). One ED reported average stays of <1 hour.

Only 13% of respondents stated that <20% of the total hospital admissions came through the ED, while 40% responded that 20-60% of admissions came through the ED. Twenty-one percent reported that >60% of admissions came through the ED. Additionally, most EDs (63%) reported having 20% or more of their visits resulting in a hospital admission.

### Capacity

**Figure 3:**
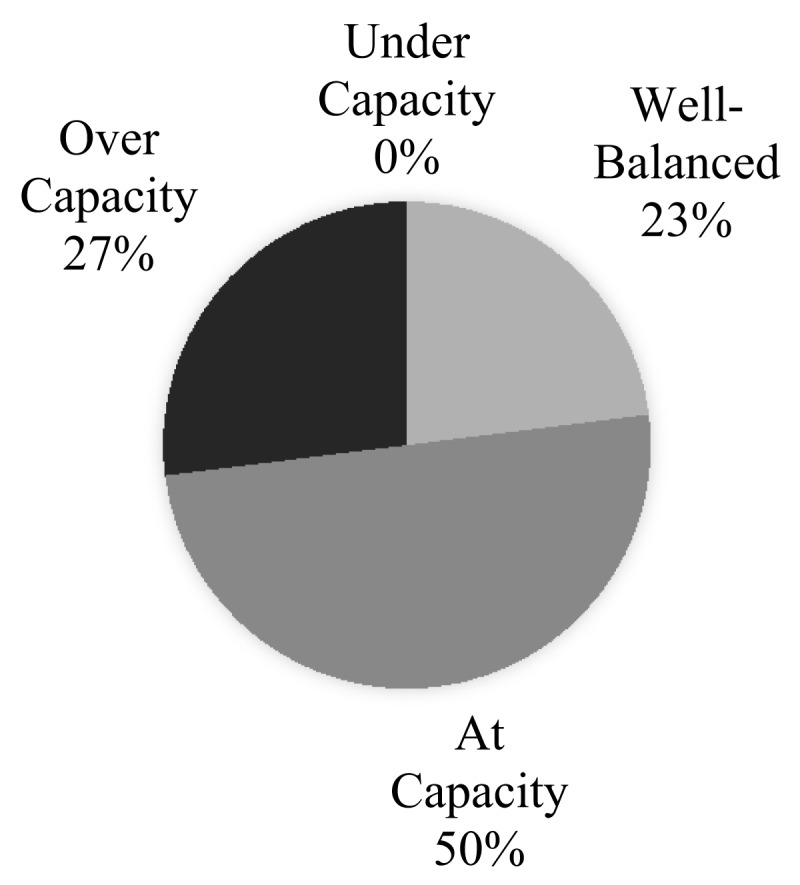
Reported Capacity of Surveyed Emergency Departments (n = 26).

Approximately 23% of respondents reported their EDs’ capacity (i.e., rooms and other resources compared to the volume of patients) to be well-balanced, while 50% and 27% reported them to be at or above capacity, respectively. Zero respondents stated that their EDs were below capacity (***Figure 3***).

### Resources and Capabilities

All respondents reported having a nurse available in the ED at all times. Physicians were also always available in all facilities, either physically in the ED 24/7 (96%) or elsewhere in the hospital and available to come to the ED 24/7 (1 ED). In terms of technological resources, most EDs reported having mechanical ventilators (96%), laboratory with 24/7 potassium measurement capability (92%), and cardiac monitoring (88%) readily available. Only 31% of EDs reported having a negative pressure room available (***Table 1***).

The majority of EDs were able to treat most types of emergencies, except for surgical hand emergencies (45%) and dental emergencies (28%, ***Table 2***). More than 85% percent of EDs were able to treat general medical, general surgical, gynecologic, and orthopedic emergencies. In terms of access to in-person, 24/7 specialist consultation, the most commonly available were: obstetrics and gynecology (91%), general surgery (87%), orthopedics (86%), and anesthesia (86%). Psychiatry (39%), cardiology (41%), and neurology (45%) where the least available (***Table 3***).

**Table 2:** Emergency Types Identified as Treatable in Surveyed Emergency Departments in Quito, Ecuador.


EMERGENCY TYPE	PERCENTAGE OF EDS ABLE TO TREAT 24/7	95% CONFIDENCE INTERVAL

Medical: general (n = 25)	92%	81–100%

Surgical: general (n = 25)	92%	81–100%

Gynecological (n = 26)	88%	76–100%

Surgical: orthopedic (n = 23)	87%	73–100%

Obstetrical (n = 25)	84%	70–98%

Toxicological (n = 24)	83%	68–98%

Trauma (n = 24)	83%	68–98%

Medical: cardiology (n = 24)	75%	58–92%

Neurological and neurosurgical (n = 22)	73%	54–91%

Surgical: plastic (n = 22)	73%	54–91%

Ear, nose, throat (n = 24)	71%	53–89%

Medical: oncology (n = 19)	68%	48–89%

Surgical: oral maxillofacial (n = 21)	67%	47–87%

Urological (n = 25)	64%	45–83%

Psychiatric (n = 19)	63%	41–85%

Ophthalmological (n = 20)	60%	39–81%

Surgical: hand (n = 22)	45%	25–66%

Dental (n = 18)	28%	81–100%


Abbreviations: ED = Emergency Department; n = number of EDs that answered question; Unknown = respondent did not know answer.

**Table 3:** Availability of Consultants in Surveyed Emergency Departments in Quito, Ecuador.


CONSULTANT DEPARTMENT	PERCENTAGE OF EDS WITH 24/7 CONSULTANT ACCESS	95% CONFIDENCE INTERVAL

OB/GYN (n = 23)	91%	80–100%

General Surgery (n = 23)	87%	73–100%

Anesthesia (n = 22)	86%	72–100%

Orthopedic Surgery (n = 22)	86%	72–100%

Neurosurgery (n = 18)	56%	33–79%

Plastic Surgery (n = 22)	55%	34–75%

Neurology (n = 22)	45%	25–66%

Cardiology (n = 22)	41%	20–61%

Psychiatry (n = 18)	39%	16–61%


Abbreviations: ED = Emergency Department; OB/GYN = Obstetrics and Gynecology; n = number of EDs that answered question; Unknown = respondent did not know answer.

### Government and Private Emergency Department Comparison

There were 12 government EDs and 14 private EDs. ***Table 4*** compares government (public and Social Security) and private EDs. Significant differences were found in mean yearly visits (50,090 government vs. 13,968 private, p < 0.001), mean number of ED beds (36 government vs. 9 private, p = 0.002), length of stay (58% of patient stays >6 hours in government EDs vs. 86% of patient stays 1–6 hours in private EDs, p = 0.009), and ability to handle cardiac emergencies 24/7 (100% government vs. 57% private, p = 0.02).

**Table 4:** Comparison Between Government and Private Emergency Departments in Quito, Ecuador.


	GOVERNMENT (n = 12)		PRIVATE (n = 14)		p-VALUE

	PROPORTION OR MEDIAN	95% CONFIDENCE INTERVAL	PROPORTION OR MEDIAN	95% CONFIDENCE INTERVAL	

**ED Characteristics**					

Contiguous	92%	56–99%	100%	–	0.46

Total Visits	50,090	31,530–87,413	13,968	6,000–20,160	**<0.001**

Percent of Pediatrics Patients	27%	14–33%	34%	29–40%	0.11

ED Beds (median)	36	18–52	9	5–18	**0.002**

Triage to a Service	64%	32–87%	50%	23–77%	0.68

**Patient Experiences in the ED**					

Percent of ED pts arriving by ambulance					0.43

<20	30%	9–64%	54%	27–79%	

20–39	10%	1–50%	23%	7–54%	

40–59	20%	5–56%	8%	1–42%	

Unknown	40%	15–72%	15%	4–47%	

Length of Stay					**0.009**

< 1 hour	0%	–	7%	1–39%	

1–6 hours	42%	18–70%	86%	55–97%	

6+ hours	58%	30–82%	7%	1–39%	

Percent of ED visits leading to admission					0.65

<20	30%	9–64%	14%	3–45%	

20–39	40%	15–72%	43%	20–70%	

40–59	10%	0–45%	29%	11–58%	

Unknown	20%	1–50%	14%	3–45%	

**Resources and Capabilities**					

Nurse in ED 24/7	100%	–	100%	–	

Physician for ED					0.99

In ED 24/7	100%	–	92%	58–99%	

In Hospital 24/7	0%	–	8%	1–42%	

On call from home 24/7	0%	–	0%	–	

Mechanical ventilator	100%	–	93%	–	0.99

24-hour lab availability	83%	52–98%	100%	–	0.2

Cardiac Monitor	83%	52–98%	93%	66–99%	0.58

EMR	42%	15–72%	100%	–	0.99

Dedicated CT	67%	35–91%	69%	39–91%	0.61

Negative pressure room	33%	10–65%	29%	8–58%	0.99

**Capacity**					0.24

Under capacity	0%	–	0%	–	

Well-balanced	25%	8–57%	21%	7–51%	

At capacity	33%	12–64%	64%	36–85%	

Over capacity	42%	18–70%	14%	3–45%	

**Treatable Medical Emergencies 24/7**					

Medical: general	100%	–	86%	55–97%	0.49

Surgical: general	91%	53–99%	93%	60–99%	0.99

Gynecological	91%	53–99%	86%	57–98%	0.99

Surgical: orthopedic	100%	–	77%	46–93%	0.23

Obstetrical	91%	53–99%	79%	49–93%	0.60

Toxicological	91%	53–99%	79%	49–93%	0.60

Trauma	100%	–	69%	39–89%	0.10

Medical: cardiology	100%	–	57%	30–80%	**0.02**

Neurological and neurosurgical	70%	36–91%	75%	43–92%	0.99

Surgical: plastic	89%	47–99%	62%	33–84%	0.33

Ear, nose, throat	82%	48–98%	62%	33–84%	0.39

Medical: oncology	67%	31–90%	70%	35–91%	0.99

Surgical: oral maxillofacial	67%	31–90%	67%	36–88%	0.99

Urological	73%	40–92%	57%	30–80%	0.68

Psychiatric	78%	40–97%	50%	21–79%	0.35

Ophthalmological	70%	39–95%	50%	21–79%	0.65

Surgical: hand	40%	15–72%	50%	23–77%	0.69

Dental	33%	10–69%	22%	5–61%	0.99

**Consultant Availability 24/7**					

OB/GYN	82%	47–96%	100%	–	0.22

General Surgery	80%	44–95%	92%	58–99%	0.56

Anesthesia	90%	50–99%	83%	50–96%	0.99

Orthopedic Surgery	100%	–	77%	46–93%	0.24

Neurosurgery	83%	33–98%	42%	17–71%	0.15

Plastic Surgery	67%	31–89%	46%	21–73%	0.42

Neurology	44%	17–76%	46%	21–73%	0.99

Cardiology	33%	10–69%	46%	21–73%	0.67

Psychiatry	22%	5–61%	56%	23–84%	0.34


## Discussion

EDs in Quito are quite diverse in size, resource availability, and capabilities. The number of ED beds ranged from 2 to as many as 61, with a median of 17; annual visits ranged from 1,600 to 129,000, with a median of ~26,000. Based on the available data, we estimate ~336 ED visits per 1,000 population in Quito, which is lower than the U.S. (433) [17] and Bogotá (569) [18], in neighboring Colombia, and higher than Switzerland (214) and Abuja, Nigeria (54) [19]. In Quito, the mean number of beds and yearly visits were significantly higher in government EDs than in private EDs. All but one ED saw children in Quito with a median of 30% pediatric visits. ***Figure 2*** displays the geographic distribution of EDs in metropolitan Quito. Although outside of the scope of this study, future studies could look at the geographic distribution of EDs in the city and socioeconomic determinants of health.

Fifty-seven percent of EDs in Quito triaged patients to a specific service, rather than caring for patients in one unified department. Information on which specific services staffed each ED was not collected. However, based on experience visiting and working in Ecuadorian EDs, typical services include pediatrics, obstetrics, internal medicine, and surgery [12]. While EM has existed as a distinct specialty in Ecuador since 1993, there are relatively few EM residency-trained physicians nationwide [12]. As this number continues to grow, it is expected that these physicians will play a larger role in the provision of emergency care within the ED.

A relatively high percentage of patients arrive to EDs in Quito via ambulance, when compared to results from other NEDI surveys, including Beijing and Bogotá. Sixty-three percent of EDs in Bogotá reported that <20% of their patients arrived via ambulance; similarly, in Beijing, 100% of EDs surveyed reported that <15% of patients arrived via ambulance [18,20]. In contrast, in Quito, only 21% of EDs reported that <20% of patients arrived via ambulance. This statistic could have been skewed by the fact that the four EDs in Quito that did not provide statistics on ambulance arrivals tended to be lower acuity facilities.

Many EDs reported long lengths of stay and crowding at their facilities. In Quito, longer lengths of stay were associated with a higher number of annual ED visits. Government EDs had significantly longer length of stays with 58% reporting mean stays longer than six hours vs. only 7% in private EDs. Multiple factors could be contributing to this difference. In general, government hospitals serve a much larger portion of the population than private hospitals (88% vs. 12%) [21]. Human and material resources could be more limited in government EDs, resulting in delays. Boarding is also a common in some government EDs and likely a major contributor to a high length of stay. Three-quarters of the facilities surveyed reported being at or over capacity. Government EDs were more likely to report being over capacity than private EDs (42% vs. 14%), but this difference did not achieve statistical significance (p = 0.24). ED crowding is a common problem that affects facilities in both developed and developing countries [22]. Researchers who conducted NEDI surveys in Bogotá and Beijing speculated that long ED lengths of stay may be secondary to ED boarding and the lack of specialist availability [18,20]. These factors may play a role in Quito as well. While this study highlights differences in length of stay and capacity between government and private hospitals, our data does not provide an explanation for these differences. Further studies will be needed to find the causes of these disparities.

The availability of resources varied across EDs in Quito. Most notably, only 31% of EDs had negative pressure rooms, in contrast to ventilators, which were available in most facilities (96%). However, no statistically significant differences were found between government and private EDs in this regard. Regionally, a greater percentage of facilities in Bogotá, Colombia, reported having a negative-pressure room [18]. Importantly, data for our study was collected before the COVID-19 pandemic, which overwhelmed Ecuador’s healthcare system [23]. Given the important role respiratory isolation can play in the containment of the virus, it is possible that the pandemic will increase awareness and implementation of this important resource.

A relatively high proportion of EDs in Quito had dedicated CT scanners (67%) when compared to Bogotá (39%) [18]. While this may represent a real difference in resource availability between countries, it is possible the current difference is much smaller. The NEDI survey in Bogotá was conducted six years ago, and it is very likely that the availability of CT scanners has increased since.

The ability to treat different types of emergencies varied amongst facilities. General medical and surgical conditions, gynecological, orthopedic, toxicological, and trauma complaints could be managed 24/7 by >80% of EDs. In contrast only 45% and 28% could handle surgical hand and dental emergencies. The ability to manage cardiac emergencies 24/7 was statistically significant difference between government and private hospitals (100% vs. 57% p = 0.02). A potential explanation for this is that private facilities tended to be smaller, and perhaps less likely to have personnel comfortable with cardiac emergencies at all times. While the initial management and stabilization of these conditions fall within the scope of practice of EM residency-trained emergency physicians, the limited availability of such physicians may impact care. Ultimately, the capacity to manage these conditions may increase as the number of EM residency-trained emergency physicians increases over time.

Overall, EDs in Quito appear to be similarly resourced, and sometimes better resourced, than EDs in other cities and countries where the NEDI survey has been performed. Based on the results of the NEDI survey, issues related to ED capacity, including length of stay and overcrowding, seem to be the most important challenges for the Quito emergency care system.

Ecuador, like the rest of Latin America and other low- and middle-income countries, is already in epidemiological transition, and emergency care is an important part of addressing its disease burden. Research from other low- and middle-income countries reflects the important role systematic emergency care can have with respect to reducing the global burden of disease. Using data from the 2010 Global Burden of Disease Study, researchers found that all 15 leading causes of death and disability-adjusted life years included potentially emergent conditions [24]. The specialty of emergency medicine has been growing in Ecuador over the last three decades but important challenges remain [12]. As the COVID-19 pandemic has highlighted, emergency care is a critical component of public health infrastructure. Greater investment in emergency departments, prehospital care, and emergency medicine personnel training could lead to stronger emergency care and public health.

### Limitations

The NEDI survey has not been validated against objective data. However, the tool has been used in more than a dozen countries and relies on reports from knowledgeable people within each ED (typically the ED director). The NEDI survey allows for comparisons across countries. To our knowledge, there is no other tool available which allows for similar comparisons to be made.

The results of this survey may also be limited by recall bias, as the respondents were asked to provide information from a prior year. We attempted to address this issue by performing a second round of data collection in which we specifically sought out information pertaining to annual visit volumes as these were not readily available or hard to estimate for respondents. Similarly, the survey format is vulnerable to social desirability bias. This was mitigated by informing respondents that results would be reported in aggregate form only and that their responses were confidential. The question about ED capacity was asked in general terms rather than providing a definition for “under capacity,” “at capacity,” or “over capacity.” This was done to make it easier for participants to respond, but it does limit comparisons across countries in this regard.

A limitation of our study is the lack of detailed information about human resources. Looking at variables such as how many nurses, general physicians, and residency-trained EM physicians work in each ED, their level of training, and their working conditions in future studies could be helpful for decision makers.

Lastly, while this study is the first publication in an indexed journal about ED capacity in Ecuador and provides a good starting point when learning about emergency care in the country, its results may not be generalizable to other Ecuadorian cities. Since Quito is the capital city and home to the only two EM residency programs in the country, it is likely better resourced than the rest of the country. The current results may represent a “best case” scenario.

## Conclusion

Little has been published about emergency care in Ecuador. While this study provides an overview of the characteristics, capabilities, and resources of emergency departments in Quito in the year 2017, many unanswered questions remain. We hope that this study serves as an important benchmark for future development work in emergency care and emergency medicine in Ecuador.

## Additional File

The additional file for this article can be found as follows:

10.5334/aogh.3129.s1 Online Supplement A. Spanish Survey Form.
