# Supplementary material for: Emergency Department Characteristics and Capabilities in Quito, Ecuador
Source: Ann Glob Health. 2021 Apr 7;87(1):37. doi: 10.5334/aogh.3129 (PMC8034401; doi:10.5334/aogh.3129)
Supplement: Online Supplement A. — Spanish Survey Form. [file agh-87-1-3129-s1.pdf]

Online Supplement A: Spanish Survey Form

Encuesta Internacional de Departamentos de Emergencias

El proyecto NEDI es conducido por la Red de Medicina de Emergencias (EMNet, grupo de investigación en Medicina de Emergencias), conformado para entender mejor las características y alcances de los Departamentos de Emergencias (DE, equivalente a Servicios de Urgencias) a nivel internacional. Ya que los problemas médicos inesperados afectan a las personas en todos los países, NEDI pretende describir los diferentes sistemas de emergencias en salud alrededor del mundo. La encuesta NEDI en linea identifica similitudes y diferencias en los DE siguiendo como filosofía en que los sistemas de emergencias en salud puedan aprender unos de otros. Usando la tecnología para facilitar la cooperación, esperamos que NEDI sirva como una plataforma de intercambio de ideas para mejorar la accesibilidad y calidad de la atención de emergencias para todos.

EL DILIGENCIAMIENTO DE ESTA ENCUESTA ES DE CARÁCTER VOLUNTARIO Y ANONIMO.

Si usted trabaja como clínico en un DE, por favor continúe con la encuesta.

¿Está dispuesto a proporcionar información descriptiva sobre su Departamento de Emergencias (Urgencias) ?

Sí
No

→ Si la respuesta es NO, por favor guarde esta encuesta y regrese a ella cuando se encuentre listo para completarla. Gracias por su tiempo.

Por favor responda las siguientes preguntas con los estimados para el año 2017 (a menos que se indique algo distinto).

1. ¿Está su DE físicamente en un hospital (por ej. En el mismo edificio o adyacente):

Sí
No

→ Si la respuesta es NO, por favor especifique la ubicación o estructura del DE (por ej. En un Centro Materno-Infantil, en un Centro de Salud independiente):

2. Especifique el número total de camas de su hospital (ingrese 999999 si se desconoce**):**

3. ¿Cuál es el porcentaje de pacientes pediátricos en su servicio? (ingrese 999999 si se desconoce**)**

4. La planta física de su DE está:

Contigua – toda la atención de urgencias médicas (por ej. infarto agudo al miocardio, asma agudo) y quirúrgicas (por ej. trauma) son proporcionadas por un DE general en un área unificada.

Ó

No-contigua – la atención de urgencias es proporcionada en áreas separadas en el hospital; ej. la atención de urgencias médicas (por ej. infarto agudo al miocardio, asma agudo) es proporcionada en un área separada geográficamente de la atención de urgencias quirúrgicas

5. ¿Existe un sistema de triage por servicio? (ej. Se realiza un triage de los pacientes a un servicio específico, Cirugía vs. Medicina)

Sí No

6. Por favor indique el número total de camas en el DE (ingrese 999999 si se desconoce)**:**
***6. Tomando en consideración las áreas de emergencia, geográficamente separadas que se relacionan en la pregunta previa ¿Cuál es el número total de camas en el DE?** Ingrese 999999 si no lo sabe**.**

7. En el 2017, ¿estuvo su DE abierto las 24 horas del día, 7 días a la semana?
*7. **En el 2017, ¿estuvo <u>por lo menos una</u> de las áreas del DE abierta 24/7?**

Sí No

8. Por favor verifique cuál de las siguientes áreas tiene su servicio e indique si hay un proveedor del cuidado de la salud disponible 24 horas al día, 7 días a la semana (24/7) en el área especificada.

DE Area	Disponible 24/7?
Trauma	
Cirugía general	
Ortopedia	
Neurocirugía	
Cirugía plástica	
Urología	
Medicina general	
Neurología	
Cardiología	
Pediatría	

Oído, Nariz, Garganta	
Oftalmología	
Obstetricia-Ginecología	
Psiquiatría	
Unidad de atencion primaria de salud/ clínica donde no hay que pedir hora para ver al médico/ unidad de cuidados primarios	
OTRO, especifique:	

9. ¿Existe un área central de triage? (ej. Un área central donde los pacientes son evaluados y luego redirigidos a una clínica específica u otra área.)

Sí No

10. ¿Quién dirige el departamento de urgencias?
***10. ¿Quién dirige las geográficamente distintas áreas de urgencias?**

Un director de urgencias supervisa todas las urgencias (ej. médicas y quirúrgicas)

* Un director de urgencias supervisa todas las áreas de urgencias (ej. medicina y cirugía)

Cada servicio tiene su propio director, sin embargo ellos en conjunto supervisan el departamento de urgencias (ej, mediante reuniones de la junta).

* Cada área tiene su propio director, sin embargo ellos en conjunto supervisan las actividades de urgencias (ej. mediante reuniones de la junta).

El servicio de urgencias comparte la misma área del hospital, pero son completamente independientes la una de la otra, cada una con su propio líder.

* Las áreas de urgencias son completamente separadas e independientes la una de la otra cada una con su propio líder

Otro, por favor especifique:

11. Por favor indique si los siguientes tipos de urgencias pueden ser tratadas en su DE. También indique si el tratamiento está disponible 24/7:
***11. Por favor indique si los siguientes tipos de emergencias pueden ser tratadas en por lo menos una de las áreas de emergencia geográficamente distinta. También indique si el tratamiento está disponible 24/7:**

	No	Sí	No Disponible 24/7	Disponible 24/7
a. Médicas – Cardiología (por ej. Arritmia, infarto agudo al miocardio)				
b. Médicas – Oncología (por ej. Fiebre y neutropenia)				
c. Médicas – Otros (por ej. Infección del tracto urinario, asma agudo)				
d. Trauma (por ej. Accidente automovilístico, herida por arma de fuego)				
e. Neurológicas y neurocirugía (por ej. Tromboembolia aguda, hemorragia intracraneal)				
f. Urológicas (por ej. Urolitiasis)				
g. Obstétricas (por ej. Complicaciones del embarazo)				
h. Ginecológicas (por ej. Quiste ovárico perforado, candidiasis vaginal)				
i. Oído, Nariz, Garganta (por ej. Epistaxis severa)				
j. Oftalmológicas (por ej. Glaucoma agudo, herida de ojo)				
k. Toxicológicas (por ej. sobredosis, envenenamiento por monóxido)				
l. Psiquiátricas (por ej. Psicosis)				
m. Dentales (por ej. Extracción de dientes)				
n. Cirugía – maxilofacial oral (por ej. Fractura de mandíbula, abscesos bucales)				
o. Cirugía – Plástica (por ej. Laceración severa de labios)				
p. Cirugía – Mano (por ej. Herida de tendón)				

q. Cirugía – Ortopédica (por ej. Fractura de huesos largos)				
r. Cirugía – General (por ej. Apendicitis aguda, neumotórax)				

12. Por favor indique el número anual de visitas de pacientes a su DE durante el 2017 (ingrese 999999 si se desconoce)**:**
***12. Por favor indique el número anual de visitas de pacientes a todas las áreas de DE durante el 2017 (**ingrese 999999 si se desconoce)**:**

Niños (por ej. Edades 0-17) = _____ ☐ Desconocido
\+ Adultos (por ej. Edades 18+) = _____ ☐ Desconocido
Total (Niños + Adultos) = _____ ☐ Desconocido

Si su DE usa una edad límite diferente para distinguir entre niños y adultos, por favor especifíquela: _____ años

13. Porcentaje aproximado de pacientes que llegaron al DE en ambulancia:
***13. Porcentaje aproximado de pacientes que llegaron a todas las áreas del DE en ambulancia:**

< 20%
20–39%
40–59%
60–79%
80% o más
Desconocido

14. ¿Requieren los pacientes ser referidos por un médico, o requieren llegar en una ambulancia, para poder recibir la atención de urgencias? (por ej. con excepciones raras, los pacientes sin una referencia no son atendidos)
***14. En <u>por lo menos una</u> de las áreas del DE ¿requieren los pacientes ser referidos por un médico, o requieren llegar en una ambulancia, para poder recibir la atención de emergencia?**

Sí No

15. ¿Cuál es el tiempo promedio que permanecen los pacientes en el área de su DE?
***15. Tomando en consideración las áreas geográficamente separadas ¿cuál es el tiempo promedio que permanecen los pacientes en el área de su DE?**

< 1 hora
1-6 horas
> 6 horas

16. ¿Hay un médico disponible en el DE, 24 horas al día, 7 días a la semana?

Sí—Hay un médico presente físicamente en el DE, 24/7
Sí—Hay un médico disponible para el DE dentro del hospital, 24/7
No—Personal variable (por ej. un médico está disponible para el DE fuera del hospital)

17. ¿Hay por lo menos una enfermera de turno en el DE, 24 horas al día, 7 días a la semana?
***17. ¿Hay por lo menos una enfermera de turno en por lo menos una de las áreas del DE, 24 horas al día, 7 días a la semana?**

Sí No

18. ¿Están los siguientes especialistas para interconsulta disponibles en persona para el DE? Los residentes o médicos en formación califican. **Si la respuesta es SI, por favor indique, en promedio, cuánto tiempo le toma al interconsultante llegar y si éste está disponible 24/7:**
***18.¿Están los siguientes especialistas para interconsulta disponibles en persona en por lo menos una de las áreas del DE?** Los residentes o médicos en formación califican. **Si la respuesta es SI, por favor indique, en promedio, cuánto tiempo le toma al interconsultante llegar y si éste está disponible 24/7:**

	No	Sí 0–29 min	Sí 30–59 min	Sí ≥60 min	No Disponible 24/7	Disponible 24/7
a. Anestesiólogo						
b. Cardiólogo						
c. Cirujano General						
d. Neurólogo						
e. Neurocirujano						
f. Obstetra-Ginecólogo						
g. Cirujano Ortopédico						
h. Cirujano Plástico						
i. Psiquiatra						

19. En el 2017, ¿cómo describiría el DE del hospital?
***19. En el 2017, tomando en cuenta las áreas de emergencia que usted eligi'o en la pregunta previa, ¿cómo describiría el DE no-contiguo de su hospital?**

Por debajo de la capacidad
Buen balance
En la capacidad
Por encima de la capacidad

20. Por favor responda "sí" o "no" a las siguientes preguntas:
***20. Por favor responda "sí" o "no"con referencia a <u>por lo menos una</u> de las áreas del DE:**

	No	Sí
a. ¿Hay un Tomógrafo dedicado al DE? (disponible preferencialmente para los pacientes del DE)		
b. ¿Hay un monitor cardiaco disponible inmediatamente en el DE?		
c. ¿Hay un respirador mecánico disponible inmediatamente en el DE?		
d. ¿Hay una sala de aislamiento respiratorio (presión negativa) disponible en el DE?		
e. ¿Es utilizado un sistema computarizado para recoger datos clínicos en el DE?		
f. ¿Existe acceso a internet en el area clínica de su DE?		
g. ¿Existe un laboratorio de análisis clínicos disponible para su DE con la capacidad de medir niveles de Potasio 24/7?		

21 Porcentaje aproximado del total de admisiones del hospital (por ej. Hospitalizaciones) que fueron admitidas a través del DE:
***21. Porcentaje aproximado del total de admisiones del hospital (por ej. Hospitalizaciones) que fueron admitidas a través de <u>todas las áreas</u> del DE:**

< 20%
20–39%
40–59%
60–79%
80% o más
Desconocido

No se aplica; el DE no es parte de un hospital

22. Porcentaje aproximado de visitas de emergencia al DE que terminan con admisión al hospital (por ej. hospitalización) incluyendo pacientes ingresados a la unidad de observación o en estado de observación (*En algunas instalaciones, una estadía corta en la "unidad de observación" o una admisión es clasificada como "estado de observación" si la estadía es menor a 24 horas):*

***22. Porcentaje aproximado de visitas de emergencia a <u>todas las áreas del DE</u> que terminan con admisión al hospital (por ej. Hospitalización) incluyendo pacientes ingresados a la unidad de observación o en estado de observación** (*En algunas instalaciones, una estadía corta en la "unidad de observación" o una admisión es clasificada como "estado de observación" si la estadía es menor a 24 horas:*

< 20%
20–39%
40–59%
60–79%
80% o más
Desconocido
No se aplica; el DE no es parte de un hospital

23. ¿Es el DE un departamento oficial dentro de la organización del hospital (por ej. independiente y separado de los Departamentos de Medicina o Cirugía)?
***23. ¿Es el DE no-contiguo un departamento oficial dentro de la organización del hospital?**

Sí No

→ Si la respuesta es NO, el DE/*DE areas es/son parte de qué departamento(s) (especifique):

Por favor indique algún comentario que nos podría ayudar a entender las respuestas de su encuesta. (por ej. Si su DE atiende a una población específica de pacientes o es un DE de especialidad):

¡Gracias por tomar el tiempo para completar este cuestionario!
